# Macrophages and Alcohol-Related Liver Inflammation

**DOI:** 10.35946/arcr.v37.2.09

**Published:** 2015

**Authors:** Cynthia Ju, Pranoti Mandrekar

**Affiliations:** Cynthia Ju, Ph.D., is a professor at the Skaggs School of Pharmacy, University of Colorado Anschutz Medical Campus, Aurora, Colorado. Pranoti Mandrekar, Ph.D., is a professor in the Department of Medicine, University of Massachusetts Medical School, Worcester, Massachusetts.

**Keywords:** Alcohol consumption, alcoholic liver disease, alcoholic liver injury, alcoholic hepatitis, alcohol-related liver inflammation, liver, immunity, innate immune response, adaptive immune response, macrophage, macrophage phenotypic switch, Kupffer cell

## Abstract

Recent studies have suggested that macrophages have a critical role in the development of alcohol-induced inflammation in the liver. To define the precise pathogenic function of these cells during alcoholic liver disease (ALD), it is extremely important to conduct extensive studies in clinical settings that further elucidate the phenotypic diversity of macrophages in the context of ALD. Research to date already has identified several characteristics of macrophages that underlie the cells’ actions, including macrophage polarization and their phenotypic diversity. Other analyses have focused on the contributions of resident versus infiltrating macrophages/monocytes, as well as on the roles of macrophage mediators, in the development of ALD. Findings point to the potential of macrophages as a therapeutic target in alcoholic liver injury. Future studies directed toward understanding how alcohol affects macrophage phenotypic switch in the liver and other tissues, whether the liver microenvironment determines macrophage function in ALD, and if targeting of macrophages alleviates alcoholic liver injury, will provide promising strategies to manage patients with alcoholic hepatitis.

Alcoholic liver disease (ALD) is a complex disease that affects millions of people worldwide and eventually can lead to liver cirrhosis and liver cancer (i.e., hepatocellular carcinoma). Aside from the direct cytotoxic and the oxidative-stress–mediated effects that alcohol and its metabolite, acetaldehyde, exert on hepatocytes, alcohol ingestion activates both the innate and adaptive immune responses in the liver. These responses involve multiple hepatic cell types, including resident macrophages, natural killer cells, natural killer T cells, lymphocytes, and neutrophils. In particular, resident macrophages in the liver, also known as Kupffer cells, are important for clearing pathogens, including bacteria, viruses, immune complexes, bacterial products called endotoxin or lipopolysaccharide (LPS), and tumor cells, from the liver (Jenne and Kubes 2013; Thomson and Knolle 2010). Research tools such as fate mapping, multifocal microscopy, transgenic/reporter mouse models, and next-generation sequencing recently have led to a better understanding of the origins, heterogeneity, and plasticity in the phenotypes and functions of macrophages and their circulating precursor cells (i.e., monocytes).

The activation of circulating monocytes and accumulation of macrophages in the liver are important pathophysiological features in patients with ALD. However, the role of hepatic macrophages in the pathogenesis of ALD has not been fully elucidated. This review will discuss some of the new findings in monocyte/macrophage biology, provide an update of the current studies on the involvement of liver macrophages in ALD, and identify remaining questions to be addressed in order to develop macrophage-targeted therapy for ALD.

## Phenotypic and Functional Heterogeneity of Monocytes and Macrophages

Macrophages, which play an important role in the initial innate immune response to infection with pathogens or other insults, fall into two main categories—infiltrating macrophages and tissue-resident macrophages. Infiltrating macrophages are derived from precursor cells called monocytes that circulate throughout the body and are recruited into the tissues when an inflammatory reaction occurs. Tissue-resident macrophages, in contrast, always remain localized within one tissue, serving as sentries and first line of defense against any infection or injury in that tissue.

Monocytes are circulating innate immune cells formed from progenitor cells in the bone marrow; the monocytes then differentiate into numerous subsets of macrophages (Fogg et al. 2006). In both humans and mice, monocytes can be divided into two major subsets—classical and nonclassical—depending on the marker proteins that they exhibit on their surface (Ingersoll et al. 2011; Sunderkotter et al. 2004; Ziegler-Heitbrock 2007) (see table 1). In humans, monocytes (all of which express CD115^+^) are divided into the two major subsets based on their CD14 and CD16 expression, as well as on their expression of markers called CCR2 and CX3/CR1:

- The predominant subset, representing 90 percent of circulating monocytes, is the classical subset characterized by the marker combination CD14^hi^CD16^−^ and CCR2^+^/CX3CR1^lo^.
- The less abundant nonclassical monocyte subset further can be divided into two groups characterized by the marker combinations CD14^dim^CD16^+^ and CCR2^−^/CX3CR1^hi^/CCR5^hi^ (nonclassical monocytes) or CD14^hi^CD16^+^ and CCR2^−^/CX3CR1^hi^/CCR5^hi^ (intermediate monocytes).

Analogous to their human counterparts, murine subsets include classical Ly6C^hi^ monocytes, which are similar to the human CD14^hi^CD16^−^ monocytes, and nonclassical Ly6C^lo^ monocytes, which are similar to human CD14^dim^CD16^+^ monocytes. These human and murine cells patrol in the blood vessels until they are recruited to the organs in case of an injury or insult. Although the gene-expression profiles related to activation and trafficking have been well conserved between murine and human monocytes, the ratios of various subsets can differ between mice and humans. Therefore, it is important to be careful and take these differences into consideration when extending experimental murine monocyte studies to human disease.^1^

Resident macrophages can be found in a variety of tissues, such as the brain, skin, lungs, liver, and spleen. Tissue-resident macrophages exhibit a large diversity of phenotypes and functions, based on their tissue of residence, raising the question of the origin of these cells (Davies et al. 2013). Recent fate-mapping studies have revealed that embryonic yolk sac and/or fetal liver progenitor cells are the source of many tissue-resident macrophages, such as those in the liver (i.e., Kupffer cells), skin, and central nervous system (i.e., microglia) (Gomez Perdiguero and Geissmann 2013). Tissue-resident macrophages are defined as a heterogeneous population of immune cells important for maintaining the homeostatic function of the specific tissue (Davies et al. 2013). Whether tissue macrophages are self-renewing or continuously replenished from the bone marrow still is a matter of debate. However, overwhelming evidence suggests that bone-marrow–derived circulating monocytes can be recruited to the site of injury early during inflammation in tissues, where they differentiate into macrophages. Classical and nonclassical monocytes are recruited in a sequential fashion, depending on the nature of insult (e.g., infection or infarction) and the injured tissue. Additionally, both resident macrophages and recruited monocytes reportedly are capable of self-renewal induced by certain cytokines, such as interleukin (IL)-4 (Jenkins et al. 2011).

The Kupffer cells in the liver are the largest population of tissue-resident macrophages and largely contribute to inflammatory reactions in the liver. The innate immune function of Kupffer cells not only is critical in the body’s response to liver injury but also is crucial in tolerogenic responses to antigens in the liver. Kupffer cells are located in the hepatic sinusoids and fall into two major subsets (Klein et al. 2007):

- Radiosensitive macrophages that are replaced rapidly by hematopoietic precursors and are important in inflammatory reactions; and
- Radioresistant, long-lived Kupffer cells that do not participate in inflammatory foci.

Mouse models frequently are used to investigate various aspects of macrophage function. However, as with the monocyte precursors, differences in the characteristics of murine and human macrophages exist that must be taken into account when using mice as preclinical models of disease (Mestas and Hughes 2004). For example, murine and human macrophages can differ in the expression of surface molecules called Toll-like receptors (TLRs) that are involved in macrophage activation, in their responses to immune activators, and in their production of nitric oxide.

## Macrophage Polarization

**Figure 1:**
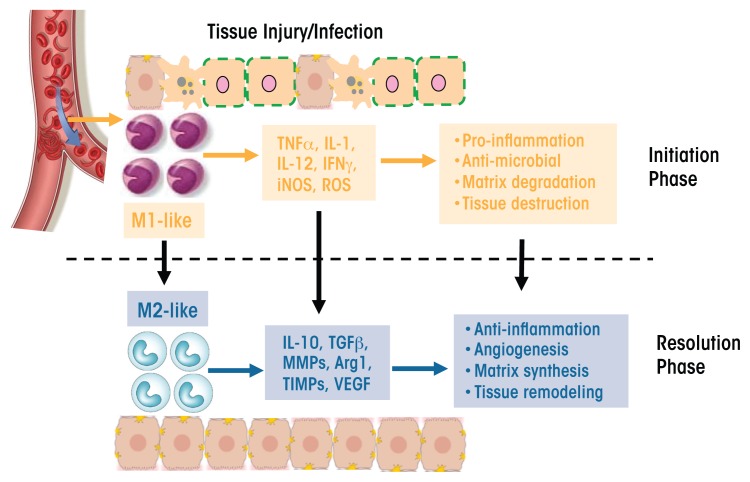
Schematic representation of macrophage plasticity and its involvement in tissue injury. Macrophages recruited to the site of an injury or infection during the initiation phase of the inflammatory reaction have an M1 phenotype. They produce proinflammatory and stress mediators and cytokines, such as tumor necrosis factor α (TNFα), interleukin (IL)-1 and -12, interferon γ (IFNγ), an enzyme generating nitric oxide (iNOS), and reactive oxygen species (ROS). These macrophages have proinflammatory and antimicrobial effects and lead to matrix degradation and tissue destruction. During the resolution phase of the injury, these M1 macrophages are converted into an M2 phenotype with a different cytokine and chemokine repertoire, including IL-10, transforming growth factor β (TGF-β), matrix metalloproteinases (MMPs), arginase 1 (Arg1), tissue inhibitors of metalloproteinases (TIMPs), and vascular epithelial growth factor (VEGF). These M2 macrophages have anti-inflammatory effects and promote blood-vessel formation (angiogenesis), matrix synthesis, and tissue remodeling.

Macrophages have a unique ability to alter their phenotypes and, thus, their functions, depending on tissue microenvironmental cues, such as the presence of cytokines, growth factors, pathogen-associated molecular pattern molecules (PAMPs), and damage-associated molecular pattern molecules (DAMPs). This process is known as polarization and results in the emergence of two macrophage phenotypes labeled M1 and M2 macrophages. M1 macrophages primarily have proinflammatory effects. For example, classically activated M1 macrophages help mediate the initial defense against intracellular bacteria and viruses; in addition, they are important for the response to a tissue injury. The M1 macrophages produce proinflammatory and stress mediators and cytokines, such as IL-1, tumor necrosis factor alpha (TNFα), interferon γ, IL-12, IL-18, nitric oxide, and reactive oxygen species (ROS), and can activate adaptive immune responses (Jouanguy et al. 1999; Shaughnessy and Swanson 2007). Once the infection or injury is controlled, macrophages convert to an anti-inflammatory, tissue-restorative phenotype in order to reign in excessive tissue-damaging inflammatory responses (Benoit et al. 2008; Noel et al. 2004). These cells usually are referred to as alternatively activated macrophages (M2) and help promote the resolution of inflammation as well as tissue repair (Sica and Mantovani 2012) (see figure 1). They can be distinguished from the M1 macrophages by the presence of high levels of several marker proteins (e.g., Fizz1, Mrc1, Ym1, and Arg1) (Gordon 2003; Mantovani et al. 2002).

The functional heterogeneity of macrophages is reflected in their differential, sometimes opposing, roles in various diseases (Sica and Mantovani 2012). For example, whereas M1 cells are essential for eliminating bacteria and viruses during acute infection, a dysregulated M1 response can result in collateral tissue damage. Thus, the proinflammatory function of M1 macrophages contributes to conditions such as autoimmune diseases (e.g., arthritis and multiple sclerosis) and metabolic diseases (e.g., insulin resistance, diabetes, and atherosclerosis). Similarly, although M2 macrophages often are associated with tissue repair and immune regulation, excessive M2 responses can contribute to chronic diseases such as atopic dermatitis, asthma, and tissue fibrosis. Additionally, diseases characterized by changes in the phenotype of the cells over time resulting from changes in the tissue environment also may be accompanied by a switch in macrophage phenotype (i.e., macrophage plasticity). For example, during early stages of cancer, tumor-associated macrophages resemble the classically activated M1 cells, which promote anti-tumor immune responses. As the tumor progresses, however, these tumor-associated macrophages switch to a regulatory phenotype that suppresses anti-tumor immunity and facilitates tumor growth (Allavena et al. 2008). As another example, adipose-tissue macrophages in nonobese individuals primarily exhibit a wound-healing phenotype, with little production of proinflammatory cytokines. In obese patients, however, the adipose-tissue macrophages switch to a proinflammatory M1-like phenotype characterized by cytokine production that leads to insulin resistance (Zeyda and Stulnig 2007).

Although it is convenient to divide macrophages into M1 and M2 cells, it is important to note that this division is oversimplified. The M1 and M2 macrophages only represent the two extremes of a full spectrum of phenotypes, and within either category there are subpopulations with different phenotypes and functions. For example, the M2 cells can be classified into at least two subtypes, wound-healing and immune-regulatory macrophages (Edwards et al. 2006). The wound-healing macrophages develop in response to the cytokines IL-4 and IL-13 that are released by various types of leukocytes. Compared with M1 macrophages, these cells produce much lower levels of proinflammatory cytokines, ROS, and nitric oxide but higher levels of molecules that promote tissue regeneration and wound healing (e.g., mannose receptors, extracellular matrix components, and factors regulating matrix remodeling). Conversely, immune-regulatory macrophages arise during late stages of the adaptive immune response or in response to stress-induced upregulation of glucocorticoids. These macrophages are characterized by the production of high levels of IL-10. Factors that induce the generation of immune-regulatory macrophages include immune complexes, prostaglandins, apoptotic cells, adenosine, histamine, and adiponectin. Unlike the wound-healing macrophages, the regulatory macrophages do not induce extracellular matrix remodeling.

The plasticity of macrophage phenotypes is controlled by various intracellular molecular mechanisms, including signaling proteins, transcription factors, and epigenetic events. For example, activation of macrophages via TLRs and interferon receptors, which induces a signaling mechanism involving a molecule called STAT1,^2^ steers their polarization toward the M1 phenotype (Qin et al. 2012). Conversely, alternative activation via IL-4/IL-13 and STAT6-mediated mechanisms generates the M2 phenotype (Daley et al. 2010; Moreno et al. 2003; Stolfi et al. 2011). Other M2-like phenotypes are induced via IL-10/STAT3 and IL-3/STAT5 signaling mechanisms (Sica and Mantovani 2012). Another important regulator of macrophage polarization is the enzyme JNK, which phosphorylates STAT6 (Shirakawa et al. 2011). Obese mice deficient in a JNK activator called MLK3 lack M1 macrophage polarization, suggesting a role for JNK in activation of the M1 phenotype (Gadang et al. 2013). IRF proteins, which modulate the transcription of certain genes, also are important regulators of macrophage polarization. For example, IRF5 activity promotes IL-12 gene transcription and is associated with an M1 phenotype, whereas repression of IRF5 induces IL-10, resulting in an M2 phenotype (Krausgruber et al. 2011). Similarly, activation of a regulatory protein complex called Notch/IRF8 leads to M1 polarization (Xu et al. 2012), whereas activation of M-CSF/IRF4 leads to M2 polarization. Another family of proteins called SOCSs also serves as essential regulators of macrophage polarization, with the specific cytokine stimulus and SOCS isoform involved determining whether the cells attain an M1 or M2 phenotype. Thus, the presence of IL-4 acting on SOCS1/STAT1 induces an M1 phenotype (Whyte et al. 2011), whereas interferon γ acting in concert with TLR can induce SOCS3/STAT3 and result in M2 macrophage polarization (Arnold et al. 2014). Various receptors located in the cells’ nucleus, such as molecules called PPARγ, PPARδ, Krupple like factor-4, and c-myc also contribute to macrophage polarization downstream of the IRF/STAT-SOCS pathway (Zhou et al. 2014). Finally, regulatory processes that affect DNA structure and gene expression without altering the DNA sequence (i.e., epigenetic mechanisms) promote the induction of an M2 phenotype and inhibit M1-characteristic genes (Banerjee et al. 2013; Satoh et al. 2010). These epigenetic regulators include such factors as histone demethylase, Jumonji D3, and microRNA let-7c.

In ALD, macrophage imprinting and polarization to M1 or M2 phenotypes is influenced by cytokine mediators in the liver. The detailed investigation of pathways activated by cytokines and stress proteins in the liver during ALD will provide insights into the polarization of resident versus infiltrating liver macrophages.

## Macrophages in ALD

### Significance of Macrophages in Clinical ALD

Macrophages seem to play a central role in ALD. In fact, recent findings suggest the coexistence and complex inter-actions of different types of macrophages in ALD (Lee et al. 2014). Thus, immunohistochemical analyses of liver samples from patients with alcoholic steatohepatitis identified macrophages that express receptors and cytokines commonly associated with M1 cells, as well as markers associated with M2 cells. Numerous other analyses have indicated that macrophage function may be clinically correlated with disease state in patients with alcoholic hepatitis and fibrosis, as follows:

- Increased macrophage numbers have been reported in both early (i.e., fatty liver) and late (i.e., hepatitis and cirrhosis) stages of ALD (Karakucuk et al. 1989), although no clear correlation exists between macrophage numbers and disease severity.
- The levels of chemokines involved in monocyte recruitment, particularly MCP-1, MIP-1α, and MIP-1β, were increased in the liver of patients with ALD (Afford et al. 1998).
- In analyses of gene-expression profiles, the expression of inflammatory genes was higher in macrophages from patients with alcohol-related cirrhosis than in macrophages from patients with Hepatitis C virus–related cirrhosis (Tapia-Abellan et al. 2012).
- Factors that imply monocyte activation, such as neopterin and leukocyte-function–associated antigen 3, were elevated in ALD patients (Luna-Casado et al. 1997).
- Circulating monocytes from ALD patients express TNFα receptors and spontaneously produce TNFα. When stimulated by LPS, they release even higher levels of TNFα (Gobejishvili et al. 2006; Zhang et al. 2001). Highly elevated TNFα levels in the blood, in turn, are associated with poorer outcomes in patients with acute alcoholic hepatitis (Bird et al. 1990). In some cases, normal levels of the anti-inflammatory cytokine IL-10 were linked to a failure to inhibit the excessive production of TNFα (Le Moine et al. 1995).
- Patients with alcoholic hepatitis and/or cirrhosis exhibit elevated levels of other cytokines (e.g., IL-6, IL-8, and IL-18) and chemokines produced by circulating monocytes and liver macrophages (Afford et al. 1998; Fisher et al. 1999). These increased cytokine levels are correlated with clinical outcomes (Khoruts et al. 1991; McClain and Cohen 1989).
- Global gene-expression profiling of liver samples from patients with alcohol-related cirrhosis demonstrated unique gene-expression patterns that differed between early and late stages of cirrhosis. Genes expressed at much higher levels in early than late stage of cirrhosis included those related to macrophage activation, proliferation, and migration (Lederer et al. 2006), emphasizing the role of macrophages in the progression of ALD.

Additional clinical studies evaluating macrophages and circulating monocytes from human patients at different stages of ALD are needed to understand the precise functional contributions of monocytes/macrophages to disease progression.

### Role of Kupffer Cells in ALD

Kupffer cells are liver-resident macrophages that are activated through the CD14/TLR4 receptor complex in response to increased intestinal translocation of LPS during prolonged alcohol consumption and which may contribute to alcohol-induced liver injury. Animal studies have revealed that acute and chronic ethanol administration are associated with signs of CD14/TLR4 activation of macrophages in the liver, including upregulation of CD14 as well as increased production of TNFα, MCP-1, and ROS (Enomoto et al. 2001). Furthermore, depletion of liver macrophages through various approaches prevented alcohol-induced liver inflammation (Koop et al. 1997; Petrasek et al. 2012), confirming that the cells are needed to induce liver injury.

Researchers have investigated how alcohol consumption may trigger Kupffer-cell activation. ROS production may be one of the mechanisms contributing to increased sensitization of Kupffer cells to LPS in the alcoholic liver (Thakur et al. 2006). During prolonged alcohol exposure, Kupffer cells produce ROS, likely mediated by induction of an enzyme involved in alcohol metabolism in the liver (i.e., cytochrome P450 2E1) (Kono et al. 2000). The crucial role of ROS production in Kupffer-cell activation also was demonstrated in studies in which rats were pretreated with an agent that inhibits an enzyme essential for ROS production (i.e., NADPH oxidase). This pretreatment normalized ROS production in alcohol-fed rats as well as reduced phosphorylation of the signaling molecule ERK1/2 and inhibited production of the proinflammatory cytokine TNFα in Kupffer cells (Kono et al. 2000; Thakur et al. 2006).

Another essential component in alcohol-mediated Kupffer-cell activation is the CD14/TLR4 receptor complex. LPS-induced activation of this receptor complex on Kupffer cells triggers downstream signaling kinases (i.e., IRAK and IKK), ultimately leading to the induction of the proinflammatory cytokines TNFα, IL-6, and MCP-1. Consistent with this model, Kupffer cells from alcohol-fed mice are sensitized to LPS and exhibit increased LPS responses, leading to higher levels of TNFα (Nagy 2003) and MCP-1 (Mandrekar et al. 2011). Enhanced expression of multiple TLRs also can contribute to ROS-mediated Kupffer-cell sensitization in the alcoholic liver (Gustot et al. 2006). Hritz and colleagues (2008) and Inokuchi and colleagues (2011) confirmed the importance of TLR4 expression on Kupffer cells and bone-marrow–derived immune cells in ALD. However, it is unclear whether liver-resident Kupffer-cell–specific TLR4 is the only TLR contributing to alcohol-mediated pathogenesis, and this issue requires further investigation using mice deficient in macrophage-specific TLR4. Nevertheless, the findings to date suggest that both alcohol-induced ROS and increased Kupffer-cell sensitization to endotoxin, which lead to enhanced proinflammatory responses, are major players in Kupffer-cell activation in ALD.

Inhibition of Kupffer-cell activation and reduction of proinflammatory cytokines—particularly inhibition of proinflammatory cytokine production by Kupffer cells—has been a major focus of efforts to alleviate ALD. For example, it may be possible to reverse Kupffer-cell sensitization by treating alcohol-exposed Kupffer-cell primary cultures with adiponectin, an anti-inflammatory adipokine (Thakur et al. 2006). Treatment with globular adiponectin prevents LPS-stimulated TNFα expression in Kupffer cells by activating the IL-10/STAT3/hemoxygenase-1 pathway and inducing M2 macrophages (Mandal et al. 2010, 2011). M2 macrophages, in turn, seem to be associated with reduced or limited liver injury, because in current drinkers with mild liver injury and steatosis, M2 macrophages are predominant, whereas patients with severe liver injury exhibit M1 macrophages (Wan et al. 2014). Another possible approach to ALD treatment may involve the desensitization of alcohol-exposed Kupffer cells by increasing IL-10 levels. The alcohol-induced decrease in IL-10 has been shown to contribute to the sensitization of macrophages, and studies in IL-10–deficient mice found increased alcohol-mediated proinflammatory cytokine production (Hill et al. 2002). Recent studies also have indicated an IL-10–mediated protective effect via activation of TLR3 in alcoholic liver (Byun et al. 2013).^3^ Selective targeting of TLR signaling pathways in Kupffer cells likely will provide better insights into the contribution of the balance between pro- and anti-inflammatory cytokine production in ALD.

### Hepatic Infiltrating Macrophages in ALD

Tissue-resident macrophages, such as Kupffer cells in the liver, not only protect against pathogens but also help nourish and maintain the cells (i.e., exert trophic functions) and ensure tissue homeostasis. However, under stress conditions caused by infection or by inflammation in the absence of infection (i.e., sterile inflammation), additional monocytes infiltrate the damaged tissue and differentiate into macrophages that help clear the pathogens, remove dead cells and cell debris, and restore tissue homeostasis. In fact, in many disease models (e.g., peritoneal inflammation) the tissue macrophages that have been described actually are derived from such infiltrating monocytes (Ghosn et al. 2010).

Studies of acute and chronic liver injuries also have demonstrated the hepatic recruitment of monocytes. For example, acute treatment of mice with carbon tetrachloride (CCl_4_), which causes liver damage, results in an influx of infiltrating macrophages that can increase the total number of hepatic macrophages tenfold (Karlmark et al. 2009). A recent study in mice with chronic CCl_4_-induced liver fibrosis demonstrated that infiltrating macrophages played an important role in the progression and regression of the fibrosis (Ramachandran et al. 2012). Similarly, in a mouse model of acetaminophen-induced liver injury, infiltrating macrophages recruited during the recovery phase contributed substantially to tissue repair (Holt et al. 2008).

Chronic alcohol-induced liver disease also is mediated and likely propagated by infiltrating immune cells, because chronic ethanol administration can cause accumulation of infiltrating macrophages in the liver of mice (Wang et al. 2014). The infiltrating macrophages consist of two subsets—Ly-6C^hi^ and Ly-6C^low^ cells—with distinct genetic profiles. The Ly-6C^low^ cells exhibit an anti-inflammatory and tissue-protective phenotype, expressing low levels of proinflammatory cytokines and high levels of anti-inflammatory molecules that may be involved in tissue repair (Arnold et al. 2007; Nahrendorf et al. 2007). Conversely, the Ly-6C^hi^ cells exhibit a proinflammatory tissue-damaging phenotype; however, upon phagocytosis of apoptotic hepatocytes, they seem to switch to a Ly-6C^low^ phenotype (Wang et al. 2014). The two subsets of infiltrating macrophages coexist and exhibit distinct, and sometimes opposite, functions in many models of inflammatory tissue injury. In a model of kidney injury, bone marrow Ly-6C^hi^ monocytes were recruited to the injured kidney, where they differentiated into functionally distinct Ly-6C^low^ cells (Lin et al. 2009). In the livers of animals with CCl_4_-induced fibrosis, Ly-6C^low^ infiltrating macrophages, which were derived from the Ly-6C^hi^ cells, were important for resolving inflammation and fibrosis and restoring tissue homeostasis (Ramachandran et al. 2012). Studies of the contribution of infiltrating-macrophage subsets in myocardial infarction also have demonstrated sequential recruitment of Ly-6C^hi^ and Ly-6C^low^ cells into the tissue. The proinflammatory Ly-6C^hi^ cells, which infiltrate the tissue during the early phase of injury, have proteolytic and phagocytic functions. At a later phase of the myocardial infarction, Ly-6C^low^ cells are recruited that possess attenuated inflammatory properties and are involved in tissue repair by promoting blood-vessel formation (i.e., angiogenesis) and activation of heart muscle cells (i.e., myofibroblasts) (Nahrendorf et al. 2007).

In humans, an increase in the number (i.e., expansion) of the nonclassical CD14^+^CD16^+^ monocytes, which correspond to the Ly-6C^low^ infiltrating macrophages, occurs in a variety of inflammatory diseases, including rheumatoid arthritis, atherosclerosis, asthma, atopic eczema, pancreatitis, and alveolar proteinosis. Nonclassical CD14^+^CD16^+^ monocytes also expand in the circulation and liver of patients with chronic liver disease, suggesting their involvement in the progression of liver inflammation and fibrogenesis (Ziegler-Heitbrock 2007).

## Macrophage Mediators in ALD

**Figure 2:**
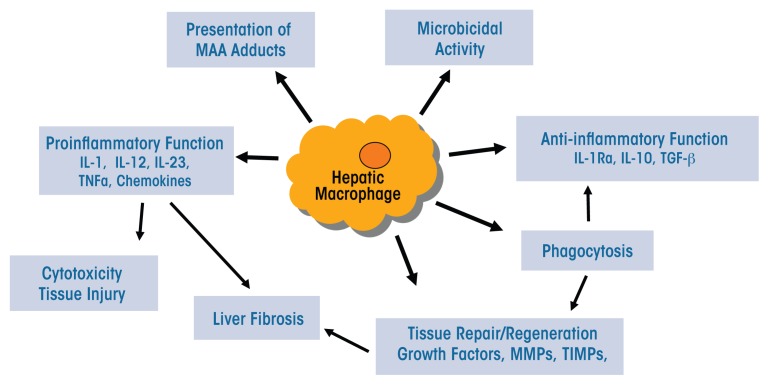
Macrophage functions in alcoholic liver disease. Macrophages fulfill a variety of functions in the context of alcoholic liver disease, including both proinflammatory and anti-inflammatory functions, depending on the state of the disease. These activities include the production of proinflammatory cytokines (e.g., interleukin [IL]-1, -12, and -23; tumor necrosis factor alpha [TNFα]) and chemokines, as well as of anti-inflammatory cytokines (e.g., IL-10, IL-1 receptor a [IL-1Ra], and transforming growth factor beta [TGF-β]). Other relevant activities include presentation of malondialdehyde-acetaldehyde (MAA) adducts and microbicidal and phagocytotic activity, as well as tissue repair and regeneration through the production of growth factors, matrix metalloproteinases (MMPs), and tissue inhibitors of metalloproteinases (TIMPs).

The heterogeneous populations of both resident and infiltrating macrophages present in the liver have multiple functions that are relevant to ALD (see figure 2):

- They can serve as antigen-presenting cells that display foreign molecules on their surface, thereby triggering adaptive immune responses.
- They may exhibit liver proteins that have been modified by malondialdehyde-acetaldehyde (i.e., malondialdehyde-acetaldehyde adducts) (Willis et al. 2002). This modification can change or impair the protein’s functions. In patients with ALD, these adducts also may be associated with the presence of autoantibodies.
- They normally produce antimicrobial peptides and mediators and have microbial killing activities; however, these functions may be compromised during ALD.
- Through activation of TLR-mediated signaling, they may lead to increased expression of immunoinhibitory receptors called PD-1 and TIM-3 on T cells, thereby impairing antimicrobial activity in patients with alcoholic hepatitis (Markwick et al. 2015).
- Certain subpopulations (e.g. Ly6C^hi^ infiltrating macrophages) produce a variety of proinflammatory mediators, including ROS, reactive nitrogen species, proinflammatory cytokines, and chemokines, thereby causing tissue damage.

Among the mediators identified, the cytokine TNFα has been extensively studied not only in patients with alcoholic hepatitis but also in animal models of ALD (Bird et al. 1990). The analyses found that mice lacking the TNFα receptor were protected from ALD (Yin et al. 1999); moreover, antibodies against TNFα were able to ameliorate alcohol-induced liver injury (Iimuro et al. 1997). Both of these findings indicate that TNFα is crucial in the pathophysiology of ALD. The role of IL-6 in ALD also has been widely investigated. Alcohol-fed, IL-6–deficient mice showed increased liver injury, suggesting a protective role for IL-6 (El-Assal et al. 2004). Additional analyses demonstrated that IL-6 reduces or increases inflammation in ALD in a cell-type–specific manner and exerts its effects via the STAT3 signaling molecule (Horiguchi et al. 2008), confirming the significant contribution of the IL-6/STAT3 axis in the development of ALD.

Other macrophage mediators involved in ALD include the chemokines IL-8, MCP-1, and MIF, which either inhibit leukocytes or help recruit them to the sites of injury and inflammation (Barnes et al. 2013; Mandrekar et al. 2011). Whereas IL-8 induces neutrophil infiltration, MCP-1 and MIF, which primarily are produced by Kupffer cells and infiltrating macrophages, facilitate the recruitment of additional monocytes/macrophages in ALD. Chemokines also induce the activation of stellate cells, which helps promote disease progression to liver fibrosis. Thus, the recruitment of inflammatory cells sets off a vicious cycle in which inflammatory and stellate cells stimulate one another, leading to fibrosis and cirrhosis (Karlmark et al. 2009). Given the central role that MCP-1 and MIF seem to play in ALD, chemokines can be considered likely therapeutic targets for this condition (Mandrekar et al. 2011; Seki et al. 2009).

Other macrophage mediators in addition to cytokines and chemokines include the complement system and adipokines such as adiponectin and leptin. The C3 and C5 complement systems are activated in macrophages during early phases of murine ALD and contribute to disease initiation and progression (Roychowdhury et al. 2009). Adipokines, in contrast, seem to negatively regulate macrophage function in murine ALD. Identification of novel macrophage mediators that can regulate polarization and thus influence development and progression in ALD is needed.

As liver injury progresses, macrophages also are needed to clear dead cells or cellular debris by phagocytosis, which is a critical step for successful resolution of inflammation and promotion of tissue repair. As a result of phagocytosis, macrophages begin to produce anti-inflammatory cytokines, such as IL-10 and TGF-β (Henson and Bratton 2013; Korns et al. 2011; Xiao et al. 2008), as well as growth factors and tissue-remodeling mediators that have proinflammatory effects. Thus, a recent immunohistochemistry study observed robust TGF-β expression in macrophages of liver samples from alcoholic hepatitis patients (Lee et al. 2014). When liver injury persists, however, the chronic inflammation and tissue-repair processes can lead to tissue fibrosis. Insufficient oxygen supply to the tissue (i.e., hypoxia) may be a factor in this process, because liver tissue hypoxia has been observed after chronic ethanol feeding (Arteel et al. 1996). Hypoxia causes stabilization and activation of proteins called hypoxia-inducible factors (HIFs), which regulate multiple pathways that control cell survival, proliferation, and metabolism. Macrophages are known to accumulate in large numbers within hypoxic areas of injured tissues (Murdoch et al. 2005). In a mouse model of liver injury, chronic liver injury induced macrophage expression of HIF1α, which promotes fibrosis by regulating the production of pro-fibrogenic mediators (Copple et al. 2012; Mehal 2012).

Oxidative-stress–mediated activation of macrophages and subsequent production of cytokines that influence macrophage polarization are major contributors to inflammation in ALD.

## ALD Therapy—Are Macrophages a Plausible Target?

Regardless of disease stage, abstinence from alcohol has been the most effective treatment in ALD. However, patients often lack motivation and compliance, leading to relapse. Another approach includes aggressive nutritional and anti-oxidant therapies using zinc (Kang and Zhou 2005), vitamins, and S-adenosylmethionine to restore nutritional status in alcoholic cirrhosis, albeit with limited beneficial outcomes. Alternative therapies using silymarin and betaine also have been suggested for future clinical trials in ALD (Frazier et al. 2011). Anti-inflammatory treatments targeting macrophage function, such as treatment with corticosteroids, pentoxyfylline, or anti-TNFα antibodies, also have been evaluated for ALD patients for more than 30 years. Success, however, has been limited to date. Clinical trials using glucocorticoids in patients with acute alcoholic hepatitis showed minor benefits but ultimately were terminated because of a heightened risk of sepsis and gastrointestinal bleeding (Maddrey et al. 1978). Subsequent studies evaluated the effects of therapy with specific anti-TNFα antibodies, again with limited success. Consequently, the need for the development of effective strategies for patients with alcoholic hepatitis and cirrhosis remains unfulfilled.

To address this need, researchers also are assessing a variety of strategies to target macrophages in preclinical murine ALD studies. These strategies often use cytokine inhibitors or intracellular mediators to regulate cytokine production, with some promising results:

- Approaches targeting alcohol-induced IL-1β signaling in macrophages using an IL-1 receptor antagonist (e.g., anakinra) have yielded a reduction in alcohol-induced inflammatory responses in murine liver (Petrasek et al. 2012).
- Studies using globular adiponectin to induce IL-10 production in Kupffer cells via the enzyme heme oxygenase-1 alleviated murine ALD (Mandal et al. 2010). Induction of this enzyme in liver macrophages by modulating carbon monoxide availability in the liver also had beneficial effects in mouse models of ALD (Bakhautdin et al. 2014).
- Efforts centering on the MCP-1 and MIF produced by Kupffer cells and infiltrating macrophages in the mouse alcoholic liver identified these chemokines as effective targets (Barnes et al. 2013; Mandrekar et al. 2011).
- Strategies targeting stress-induced heat-shock protein 90 with specific inhibitors—an approach currently assessed in clinical trials for cancer—helped ameliorate ALD by inhibiting macrophage inflammatory responses in murine liver (Ambade et al. 2014).

These studies collectively support clinical evaluations of macrophage-targeting therapies in alcoholic-hepatitis patients. Clinical research combining biologics, small-molecule drugs, and antioxidant therapies targeting macrophage function and phenotype may provide lasting therapeutic efficacy in alcoholic hepatitis and cirrhosis.

## Conclusion and Perspectives

As in many chronic inflammatory diseases, macrophages have emerged as critical players and perhaps a therapeutic target in ALD. However, depleting all hepatic macrophages will not be an effective approach because of the heterogeneity and phenotype diversity of these cells; the specific populations to be targeted for maximum benefit remain to be determined.

**Glossary**

Adipokine: A bioactive factor produced and secreted by fat (adipose) tissue that can modulate the function of other tissues
Alveolar proteinosis: A chronic lung disease characterized by the filling of the *alveoli* with a protein-like material that prevents ventilation of the affected area; results in shortness of breath, coughing, chest pain, weight loss, and spitting up of blood
Alveoli: Sac-like structures in the lungs where the gas exchange between the inhaled air and blood takes place
Autoantibody: An immune molecule (i.e., antibody) formed in response to, and acting against, one of the individual’s own normal tissue constituents
Chemokine: Any of a family of small *cytokines* that induce the movement of *leukocytes* (e.g., to the site of an infection)
Complement system: A complex system of about 20 distinct proteins, their receptors, and related regulatory proteins that induce the destruction (i.e., lysis) of cells during an immune response as well as regulate various other biologic functions (e.g., *phagocytosis*)
Cytokine: Any of the non-antibody proteins released by one type of immune cell on contact with a specific antigen that acts as a mediator between cells (e.g., in the generation of an immune response)
Damage-associated molecular pattern molecules (DAMPs): Molecules that can initiate an immune response in response to cell or tissue damage (i.e., as part of a noninfectious inflammatory response)
Endotoxin: Toxic molecule associated with the membranes of certain bacteria that are released when the cells are disrupted and have numerous biologic effects (e.g., fever, altered resistance to bacterial infection, shock); endotoxins are composed of lipopolysaccharides (LPS)
Epigenetic: Pertaining to mechanisms that alter the activity of genes without changing their DNA sequences (e.g., by chemically modifying the DNA or altering the accessibility of the DNA for regulatory proteins)
Fate mapping: An experimental approach to determine the origins of various tissues in the adult organism from the embryonic structures and to track the development of specific cells through several developmental stages
Leukocytes: Any of variety of white blood cells, such as monocytes or lymphocytes
Macrophage polarization: Process during which macrophages acquire specific characteristics and functions in response to external signals (e.g., certain *cytokines*); the two main types of polarized macrophages are M1 (classically activated) and M2 (alternatively activated) macrophages, each of which produces specific *cytokines* and induces specific immune responses
Malondialdehyde: An organic compound formed during the degradation of lipids by reactive oxygen species (e.g., during alcohol metabolism, which results in formation of reactive oxygen species); malondialdehyde can interact with certain DNA building blocks, forming DNA adducts, which can induce mutations in the DNA
Pathogen-associated molecular pattern molecules (PAMPs): Molecules that can initiate an immune response in response to infection with a pathogen (i.e., as part of an infectious inflammatory response)
Phagocytosis: Process by which a cell (e.g., a macrophage) takes up microorganisms or cell fragments in membrane-enclosed vesicles in which the engulfed material is killed and digested
Steatohepatitis: Condition in which fat droplets accumulate in the liver (e.g., as a consequence of alcohol misuse) with simultaneous inflammation of the liver
Stellate cell: Cell type found in the liver with a characteristic star-like shape that is mainly responsible for fat storage in the liver as well as for collagen production; source of excess collagen produced during hepatitis
Tolerogenic: Capable of inducing immunologic tolerance (i.e., lack of a reaction to a molecule that would normally trigger an immune response)

There are many questions that still need to be addressed with regard to the role of hepatic macrophages in ALD development. For example, how do infiltrating monocytes differentiate within the liver during ALD? What are the tissue-environmental cues and molecular-signaling pathways that drive the reprogramming of infiltrating macrophages in the alcoholic liver? Another important question is how the number of infiltrating macrophages is controlled after tissue homeostasis is reestablished. Do excess cells undergo apoptosis or do they emigrate? Moreover, in-depth knowledge of the molecules and pathways that control and regulate the phenotype and functions of hepatic macrophages is critical for developing therapeutic strategies to treat ALD. For example, the functions of Ly-6C^low^ infiltrating macrophages in tissue repair and wound healing can be utilized to prevent chronic liver inflammation during the early phase of ALD. The conversion of the proinflammatory tissue-damaging Ly-6C^hi^ infiltrating macrophages to anti-inflammatory tissue restorative Ly-6C^low^ cells can serve as a target for treatment of advanced stages of ALD, such as alcoholic steatohepatitis, and has been suggested in human and mouse studies (Singal et al. 2013). Inhibition of macrophage-mediated inflammation is already being used as a therapeutic option in other conditions; for example, agents such as statins, thiazolidinedione, and n-3 fatty acids, which can prevent macrophage-mediated inflammation, are a preferred strategy in diabetes treatment (Ji et al. 2009; Methe et al. 2005; Ramirez et al. 2008; Yeop Han et al. 2010). These therapies also warrant evaluation for their effects in attenuating liver injury and inflammation in alcoholic steatohepatitis.

## Figures and Tables

**Table 1:** Monocyte Populations of Human and Mouse Origin

Monocytes		Markers	Function
Human	Classical	CD14^hi^CD16^−^CCR2^+^CX3CR1^lo^	Phagocytosis and inflammatory effectors
	Intermediate	CD14^hi^CD16^+^CCR2^−^CX3CR1^hi^	Inflammatory effectors
	Nonclassical	CD14^dim^CD16^+^CCR2^−^CX3CR1^hi^	Patrolling, antiviral role
Mouse	Classical	CD11b^+^Ly6C^hi^CCR2^+^CX3CR1^−^	Inflammatory effectors
	Nonclassical	CD11b^+^Ly6C^lo^CCR2^−^CX3CR1^+^	Patrolling, tissue repair

**Table 2:** Complete Names of Enzymes and Other Molecules Mentioned in This Article and Their Abbreviations

Abbreviation	Complete Name
CCR2	C-C chemokine receptor 2
CD	Cluster of differentiation
CX3/CR1	C-X3-C motif chemokine receptor 1
ERK	Extracellular-signal–regulated kinase
IKK	Inhibitor of nuclear factor kappa-B kinase
IL	Interleukin
IRAK	Interleukin-1 receptor-associated kinase
IRF	Interferon regulatory factor
JNK	C-jun N-terminal kinase
LPS	Lipopolysaccharide
MCP	Monocyte chemoattractant protein
M-CSF	Macrophage colony-stimulating factor
MIP	Macrophage inflammatory protein
MLK	Mixed lineage kinase
PD-1	Programmed cell death protein 1
PPAR	Peroxisome proliferator-activated receptor
SOCS	Suppressor of cytokine signaling
STAT	Signal transducers and activators of transcription
TGF	Transforming growth factor
TIM-3	T-cell immunoglobulin mucin-3
TLR	Toll-like receptor
TNFα	Tumor necrosis factor alpha

## Footnotes

1 Comprehensive transcriptomic analysis on unique human and murine immune-cell gene expression during differentiation, activation, and tissue-specific localization is available from project consortia such as ImmGen (www.immgen.org) and InnateDB (www.innatedb.ca).

2 For a list of the complete names of this and other molecules mentioned in this article, see table 2.

3 In addition to TLR3 and TLR4, increased expression of TLR2 and TLR8 has been identified by immunohistochemistry in liver biopsies from alcoholic hepatitis patients (Lee et al. 2014).
